# Evaluation of microhaplotypes in forensic kinship analysis from a Swedish population perspective

**DOI:** 10.1007/s00414-021-02509-y

**Published:** 2021-01-28

**Authors:** Adam Staadig, Andreas Tillmar

**Affiliations:** 1 Department of Forensic Genetics and Forensic Toxicology, National Board of Forensic Medicine, Linköping, Sweden; 2 Department of Biomedical and Clinical Sciences, Division of Molecular Medicine and Virology; Faculty of Medicine and Health Sciences, Linköping University, Linköping, Sweden

**Keywords:** Microhaplotypes, Forensic genetics, Population genetics, Kinship analysis, Human identification, Genetic marker

## Abstract

The development of massively parallel sequencing (MPS) technology has enabled the discovery of several new types of forensic markers where microhaplotypes are one of these promising novel genetic markers. Microhaplotypes are, commonly, less than 300 nucleotides in length and consist of two or more closely linked single-nucleotide polymorphisms (SNPs). In this study, we have examined a custom-made QIAseq Microhaplotype panel (Qiagen), including 45 different microhaplotype loci. DNA libraries were prepared according to the GeneRead DNAseq Targeted Panels V2 library preparation workflow (Qiagen) and sequenced on a MiSeq FGx instrument (Verogen). We evaluated the performance of the panel based on 75 samples of Swedish origin and haplotype frequencies were established. We performed sensitivity studies and could detect haplotypes at input amounts down to 0.8 ng. We also studied mixture samples with two contributors for which haplotypes, for the minor contributor, were detectable down to the level of 1:100. Furthermore, we executed kinship simulations to evaluate the usefulness of this panel in kinship analysis. The results showed that both paternity and full sibling cases can clearly be solved. When simulating a half sibling versus unrelated case scenario, there were, however, some overlap of the likelihood ratio distributions potentially resulting in inconclusiveness. To conclude, the results of this initial study are promising for further implementation of this microhaplotype assay into the forensic field, although we noticed some primer design issues that could be optimized, which possibly would increase the power of the assay.

**Supplementary Information:**

The online version contains supplementary material available at 10.1007/s00414-021-02509-y.

## Introduction

The forensic field is in a continuous need of improvements due to the various and complex issues in forensic investigations, in addition with its often-intricate sample material and quality. Standard short tandem repeat (STR) analysis has limitations when working with highly degraded samples due to their relatively long fragment length. There has been an extensive technological development in the forensic field during the recent years, mainly based on the expansion of massively parallel sequencing (MPS) technology. Different applications of MPS into forensics have shown to be very successful for solving previously unsolved cases with its increased sensitivity and precision [1]. The use of MPS has enabled the detection of various new types of forensic markers, other than traditional STR markers, to gain more genetic information. One of these novel genetic markers are microhaplotypes which are, commonly, less than 300 nucleotides in length and could therefore be covered within a single sequencing read [2]. They consist of two or more closely linked single-nucleotide polymorphisms (SNPs) and the allelic combination of the linked SNPs results in the haplotype of that marker. Earlier studies have proposed these microhaplotype regions to be lineage informative and can be used in kinship analysis [2–4]. One advantage of microhaplotypes over STRs is the lack of repetitive regions that can result in stutter artefacts caused by DNA polymerase slippage during amplification. The removal of the stutter phenomenon could enhance data interpretation, especially in DNA mixture samples. The short distance between the SNPs implies a low recombination rate and each microhaplotype is considered to be inherited as a block that is being passed over from generation to generation [4]. It is also known that the mutation rates among SNPs [5] are much lower than for STR markers [6, 7]. All these features make microhaplotypes a suitable marker of choice in missing person identification where the reference individual is a close relative. Additionally, in human identification cases, the sample material can often be degraded and fragmented, wherefore the relatively long STR markers can result in incomplete DNA typing and the shorter microhaplotypes are believed to be a more appropriate marker. Microhaplotypes could also be a useful tool for predicting the biogeographic ancestry of an individual, which could be an important investigative lead in criminal investigations [2].

One drawback with SNP markers in comparison with STRs is that more markers are required to gain the same information level due to the biallelic composition of SNPs. An increased number of markers require a more careful primer design to avoid non-specific primer binding or primer dimers. However, previous studies have shown that on a per locus basis, the closely linked SNPs forming a haplotype will gain more information than single SNPs [4, 8].

One measurement to evaluate the potential of identified microhaplotypes is to calculate the effective number of alleles (*A*_e_). *A*_e_ is defined as the number of equally frequent alleles and calculated as the reciprocal of the homozygosity [9]. This value can be used as a tool to rank different microhaplotypes when selecting as informative markers as possible for the given purpose. *A*_e_ is a very effectful measurement when selecting microhaplotypes for mixture deconvolution [9]; however, it has also been shown to have an impact when selecting for lineage informative markers [10]. Heterozygosity is another value that can be used to address the informative value of a locus in kinship analysis [11].

The aim of this study was to evaluate a custom-made QIAseq Microhaplotype panel (Qiagen) in a broad context for different forensic applications. The project was therefore divided into five different subprojects referred to as population analysis, mixture analysis, sensitivity analysis, bone sample analysis and kinship analysis.

## Materials and methods

All samples were handled and analysed according to the National Board of Forensic Medicine’s approved policy [12] and to the ethical approval by the regional ethical review board (98267).

### Library preparation and sequencing

The analyses were performed in five different runs and twenty-four samples were pooled in each sequencing run. Each run included a positive control (2800 M, Promega) and a negative control. All samples were analysed with a custom-made microhaplotype panel (Qiagen) consisting of 45 different microhaplotype markers. Construction of DNA libraries was performed using the GeneRead DNAseq Targeted Panels V2 library preparation workflow (Qiagen) [13]. Eight microliters of extracted DNA was added, and the initial step was a PCR amplification with the customized primer set. The PCR program was designed as described in the manual with 23 number of cycles (24 for the bone samples) [13]. The PCR product was then purified based on magnetic beads purification with AMPure XP beads (Beckman Coulter). The next steps were end repair of the DNA, A-addition and barcode tagging using the GeneRead Adapter I (Qiagen) enabling sample multiplexing. A clean-up of adaptor-ligated DNA was performed and followed by a second PCR with 10 cycles and a final clean-up. Quality control of the DNA libraries was done by quantification with Qubit dsDNA BR assay on a Qubit2.0 (Invitrogen) [14]. Also, the average library size was checked as a quality control step with Agilent’s High Sensitivity DNA kit on a Bioanalyzer (Agilent) [15]. The libraries were then diluted to 10 picomolar and pooled together for sequencing on a MiSeq FGx instrument (Verogen) [16].

### Bioinformatic data analysis

The generated FASTQ files from the sequencing were used as input to Biomedical Genomics Workbench 5 (CLC Bio, Qiagen) for calling of the microhaplotypes. A Qiagen custom-made workflow was used to extract the microhaplotypes from the FASTQ files. All samples were demultiplexed and the reads were mapped to a reference genome (GRCh37, hg19). Primers were trimmed off with the “*Trim primers of Mapped Paired End Reads*” function. The mapped and trimmed reads were then realigned via the “*Local Realignment function*” and SNP variants were called and microhaplotypes were assigned according to the “*Micro Haplotyping*” function. The coverage for each of the analysed microhaplotype markers was evaluated and a manually user-defined read coverage threshold was set to 200 for haplotype calling. Furthermore, haplotype read frequencies (HRF) for all markers were calculated as the read coverage from the allele with the highest read count divided by the total read count for that marker. The HRF was calculated for quality control reasons of the heterozygote balance. The read coverage selection and HRF calculations were performed in an in-house written R-script [17].

### Microhaplotype loci

Forty-five microhaplotypes (Supplementary file 1) were included in the panel and all regions were previously described by Kidd et al. [2, 4, 18] in ALFRED [19] as known polymorphic microhaplotype regions. Eight of the microhaplotype markers included in this panel correspond completely to previously reported markers in ALFRED. However, thirty-seven of the markers lacked one or more SNPs compared to ALFRED, mainly because of issues with the primer design to cover all known SNPs in one fragment at some of the regions. We did, however, identified fifteen SNPs from fourteen regions in this examined panel that were not included in ALFRED. Supplementary file 1 summarizes the observed microhaplotypes and their correspondence to previously reported microhaplotypes in ALFRED.

### Population analysis

The population analysis included blood samples from 75 individuals of Swedish origin and DNA was extracted as previously described [20]. Haplotype frequencies for the 75 population samples were estimated. A population comparison was performed with the observed microhaplotype data from this study and previously reported data in the ALFRED database by Kidd et al. [19]. Relevant allele frequencies from ALFRED were merged to be comparable with the observed SNPs in this panel. Also, haplotype frequencies from this study that contained SNPs not reported in ALFRED were merged for the same purpose. A comparison with four different populations was done, Danish [21], Finnish, Han (Chinese) and Luhya (African) populations [22]. Pairwise *F*_ST_ values were calculated in Arlequin [23] with 10,000 permutations and a significance level of 0.05. In addition, an exact test of population differentiation was performed with a significance level of 0.05.

### Bone sample analysis

Five different bone samples were randomly selected. One bone sample was degraded and the other four were not degraded. All samples were previously typed with complete STR profiles. DNA was extracted using a phenol/chloroform extraction method [24]. An increase in the number of PCR cycles for degraded samples, such as bone samples, has previously been shown successfully [25]. Therefore, this action was applied for the amplification of all bone samples and the number of cycles was increased by one cycle to 24, compared to 23 according to the manual [13].

### Sensitivity analysis

For the sensitivity study, a dilution series of the control DNA 2800M (Promega) was prepared with the following input amounts: 16 ng, 6.4 ng, 3.2 ng, 1.6 ng and 0.8 ng. Eight microliters of each DNA sample was added and the proportions of inconclusive and incorrect haplotypes were observed.

### Mixture analysis

Two different control DNA samples: 2800M (Promega) and DNA 007 (Thermo Fisher Scientific), were mixed in six different ratios: 1:1, 1:3, 1:10, 1:50, 1:100 and 1:1000 with a total DNA input amount of 16 ng. One representative microhaplotype locus (MH05) was further evaluated for the mixed samples and sequence read ratios were established.

### Kinship analysis

Extracted DNA from blood samples from individuals in two different families with known relatedness were analysed. Samples from the mother, father and their three biological children were analysed in the two families, respectively. Likelihood ratio (LR) calculations based on the generated DNA data were performed in Familias [26]. Paternity tests for all three children were performed in both duo and trio cases. Also, maternity tests for all children were performed in duo cases. We compared the hypothesis that each parent is the biological parent of each child versus being unrelated.

To evaluate the discrimination power of the panel, 10,000 simulations were performed in Familias. The following hypotheses were compared:

- H1: The alleged father is the biological father of the child.
- H2: The alleged father and the child are unrelated.

The simulations were based on the generated haplotype frequency data from the population analysis. The results were used to identify the average number of genetic inconsistencies when the alternative hypothesis (H2) was true. Furthermore, 10,000 new simulations for three different relationships were performed in ILIR [27]. The tested hypotheses were:

- Paternity trio (H1) versus maternity duo (H2)
- Two tested individuals being full siblings (H1) versus being unrelated (H2)
- Two tested individuals being half siblings (H1) versus being unrelated (H2)

### Statistical parameters

Hardy-Weinberg equilibrium (HWE) was tested in the Arlequin software [23] with the exact test settings and the number of steps in the Markov chain was 1,000,000 and the number of dememorization steps was 100,000. Also, linkage disequilibrium (LD) between all pairs of loci (946 pairwise comparisons) was tested in Arlequin with 10,000 number of permutations. Additionally, the effective number of alleles (*A*_e_) was calculated as the reciprocal of the homozygosity. Also, the heterozygosity for each locus was estimated.

## Results and discussion

### Quality control

All positive controls (2800M, Promega) displayed the same haplotypes from the five different library preparations and sequencing runs. The negative controls displayed a median read coverage of 26 reads and no read coverage above the 200 reads threshold in four of the analysis. However, in one run, two markers had read counts above the defined threshold. Locus MH12 displayed 343 reads and locus MH33 generated 603 reads.

### Population analysis

#### Read coverage

**Fig. 1:**
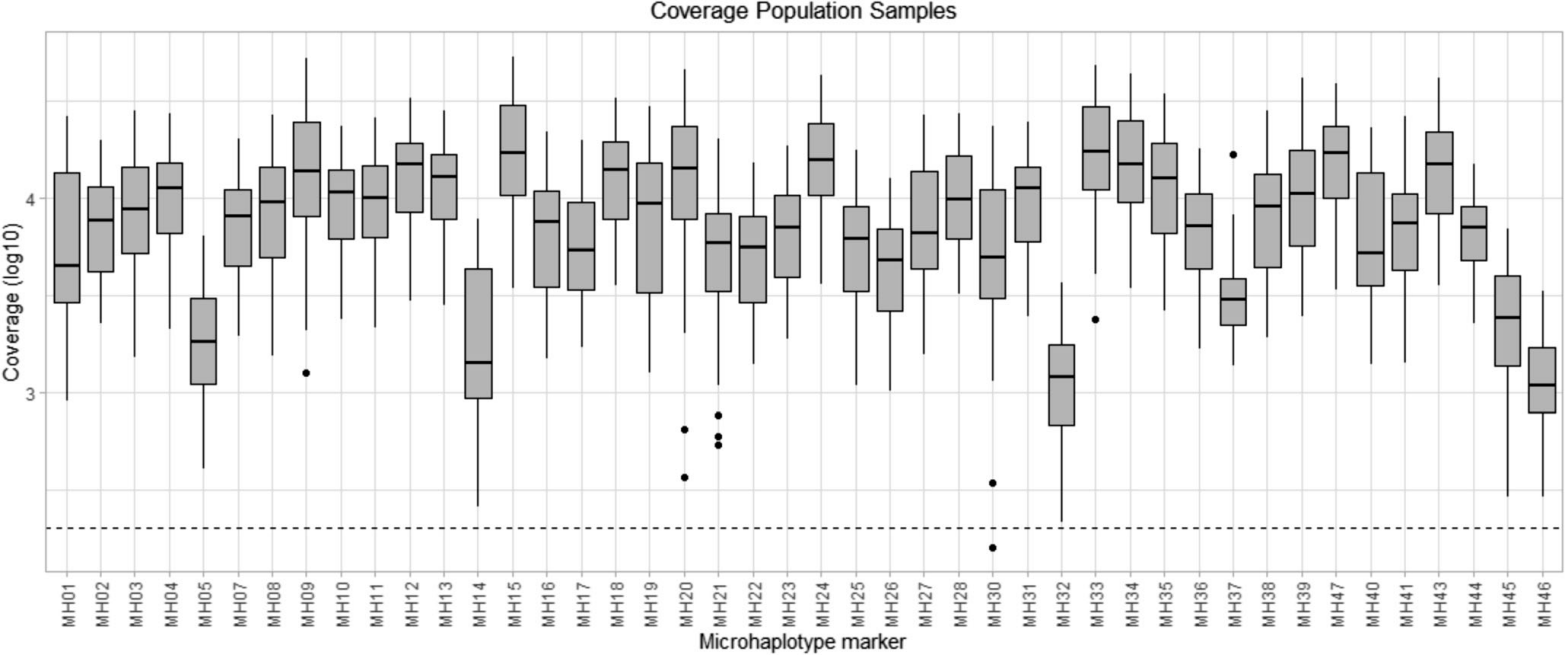
The read coverage (log10) for the 75 population samples are illustrated as a boxplot and the dashed line at the bottom represents the read coverage threshold of 200 reads. All samples are well above the threshold and are quite well balanced among each other

The results from the 75 population samples were used to evaluate the performance of each microhaplotype loci included in the panel. A coverage threshold for an allele to be called was set to 200 reads. One of the analysed markers (MH29) did not meet this criterion for most of the samples and was therefore discarded from further evaluation and analysis. All the other 44 markers were well above this threshold and were fairly well balanced among each other. This is illustrated in Fig. 1, where the read coverage per marker is presented along with a dashed read threshold line. The 44 markers that passed this quality control step were further analysed and evaluated in the study.

An initial bioinformatic analysis without any primer trimming was performed. This revealed that some markers displayed more than two alleles per individual and locus. This was caused by the primer design of the panel. In 16 of the microhaplotype regions, primers were designed with more than one primer pair per region to cover the selected SNPs. This procedure can be applicable in standard SNP analysis to cover all SNPs that are closely positioned. This approach could also be useful in microhaplotype assays if both primer pairs are positioned outside the region of interest. Unfortunately, this was not the case in this panel. The two primer pairs did not cover the whole region independently. Instead, the primers were included within the region of interest and in addition, covering some SNP variants. This primer design resulted in two partly overlapping amplicons for the same microhaplotype region, which resulted in that one could not determine which allelic combination that originates from one read, i.e. the same haplotype, since there is an overlap of the sequencing read for the region.

The bioinformatic analysis had to be optimized to solve this problem with a multiallelic appearance. Some primer pairs that were partly overlapping were excluded so that only one pair of primers covered the microhaplotype region. Unfortunately, this resulted in that one or two SNPs were excluded in some regions since these single fragments did not cover all SNPs in the microhaplotype region. See supplementary file 1 for a list of markers and SNPs that were affected and excluded. Due to this exclusion of primers, four of the markers now display only one SNP variant. As a result of this bioinformatic optimization, an average of 30% of the sequencing reads that originate from the partly overlapping fragments from the relevant markers had to be excluded. An optimization of the primers could, therefore, increase the number of reads, or clusters on the flow cell, for the relevant loci.

#### Haplotype frequencies

Haplotype data from the 75 Swedish population samples were used to establish haplotype frequencies for the 44 markers (see Supplementary file 2). The number of SNPs per microhaplotype marker ranged from one to four and the number of observed haplotypes ranged from two to eight, which are specified in Supplementary file 1.

#### HRF

**Fig. 2:**
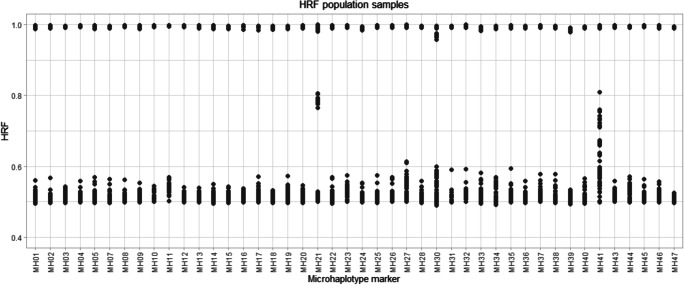
The haplotype read frequency (HRF) is presented for each of the 75 analysed population samples. Two markers, MH21 and MH41, display an imbalanced pattern

Haplotype read frequency (HRF) was calculated for each sample at each microhaplotype marker. HRF is a value used to examine the intralocus balance of a marker. HRF was calculated by taking the number of reads of the haplotype with most reads divided with the total number of reads observed for the marker. Theoretically, the HRF for a homozygote genotype would be 1 and for a heterozygote genotype 0.5. Figure 2 illustrates the HRF for each marker where each dot represents a sample. As shown, MH21 and MH41 demonstrate an imbalanced pattern for some samples. MH21 corresponds to the marker named mh11KK-036 reported by Kidd et al. [2, 4], although in this panel only one SNP is represented due to the primer design which makes it problematic to compare the marker between different studies. MH41 corresponds to mh21KK-315 reported by Kidd et al. [18], but even the SNPs from this marker do not completely overlap with the Kidd marker. We have not noticed any imbalanced pattern for the previously described markers mh11KK-036 or mh21KK-315 in the literature which indicates that the reason for the intralocus imbalance in this study most likely is due to the primer design in this assay.

#### Population comparison

An average *F*_ST_ value for each of the examined comparisons is presented in Table 1. The geographically closely located populations of Sweden, Denmark and Finland displayed, as expected, a low *F*_ST_ value which implies that they are closely related at a population level. Also, when comparing more geographically distant populations as the African Luhya population and the Chinese Han population, the observed *F*_ST_ values were higher which implies that the Swedish population is more genetically separated from those two.

**Table 1:** The average *F*_*ST*_ value for each of the tested population comparisons is presented. The Scandinavian populations display a low *F*_ST_ value and the two other populations, which are more geographically distant, shows as expected a higher *F*_ST_ value

Population comparison	Average *F*_ST_ value
Swedish vs Danish	0.0015
Swedish vs Finnish	0.0023
Swedish vs Luhya	0.099
Swedish vs Han	0.096

Moreover, the population differentiation test showed that there was no significant difference between the Swedish and Danish populations in all except one marker (MH30). There was no significant difference between the Swedish and Finnish populations in all except two markers (MH27 and MH30). For the Swedish and Luhya population comparison, there was a significant difference in all but seven markers (MH09, MH12, MH18, MH21, MH37, MH43 and MH46). In addition, the test showed a significant difference in all but 11 markers (MH02, MH09, MH18, MH21, MH24, MH26, MH27, MH35, MH37, MH43 and MH45) for the Swedish and Han population comparison.

These population comparison results showed that there was no significant difference between the Scandinavian populations. The frequency data from these populations could therefore be combined into one single reference dataset. There was, however, a notable population difference among the Swedish, Luhya and Han populations, respectively.

### Bone sample analysis

Complete haplotype profiles were observed for all examined loci in four of the analysed bone samples. Four markers in one sample did not meet the read coverage criteria and were therefore not typed. The average coverage per sample was on a median 1.9 times higher than for the population samples. A plausible explanation for this could be that the DNA library amount of the bone samples was higher than most of the other samples. Although, all samples were diluted to the same concentration prior to sequencing, there could be some issues during normalization or quantification causing this appearance. However, blood samples and positive controls were prepared and sequenced together with the bone samples and those samples did not display any increase in read counts. The read coverage (log10) is illustrated in Supplementary file 3 as a boxplot with a dashed horizontal line representing the defined read coverage threshold of 200 reads. The coverage of the markers was relatively balanced, although some outliers for specific samples exist. However, one should take into account that these results were based on the analysis of only five bone samples. The HRF was also fairly balanced, although two samples showed heterozygotic imbalance at locus MH27 (see Supplementary file 4).

### Sensitivity analysis

**Fig. 3:**
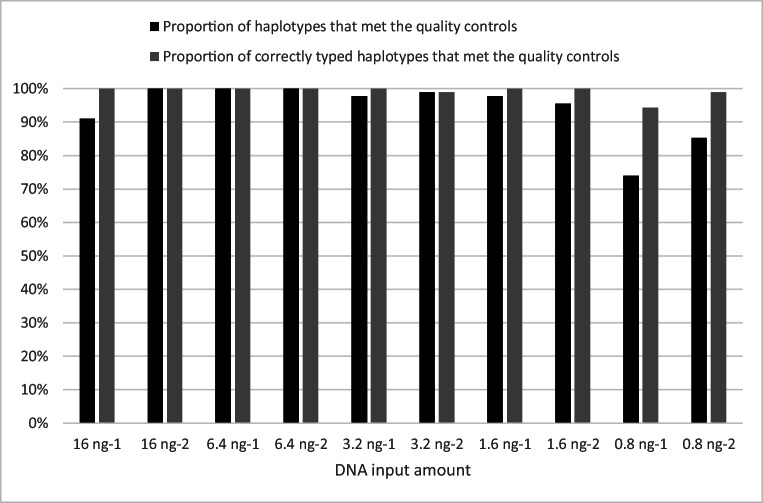
The proportion of haplotypes that met the above-defined quality controls (black bars) is presented together with the proportion of correctly typed haplotypes that met the quality criteria (grey bars). Only a few dropouts were seen down to 1.6 ng. However, the lowest input amount showed a notable number of dropouts resulting in both complete locus dropouts and false homozygotes

The sensitivity analysis was performed with five different DNA input amounts of DNA control 2800M (Promega). Each input amount was analysed in duplicate. Complete and accurate haplotypes were detected down to 6.4 ng of input DNA. Except for one replicate of the 16-ng sample where four markers (MH09, MH20, MH30 and MH45) did not meet the quality control criteria and were therefore not typed. Figure 3 illustrates the proportion of haplotypes that met the quality control criteria and the proportion of those that was correctly typed. In total, among all samples, the dropouts resulted in 23 complete marker dropouts (MH05, MH09, MH14, MH16, MH17, MH20, MH30, MH32, MH37, MH45 and MH46) and seven false homozygotes (MH7, MH23, MH32, MH36, MH41 and MH44). The majority of these dropouts resulted from the 0.8-ng samples. The false homozygotes appear in three samples as illustrated in Fig. 3 as incorrect genotype calls, since one of the alleles meet the quality controls. This appearance of haplotype or complete marker dropouts is expected since the input amount is decreased.

### Mixture analysis

**Fig. 4:**
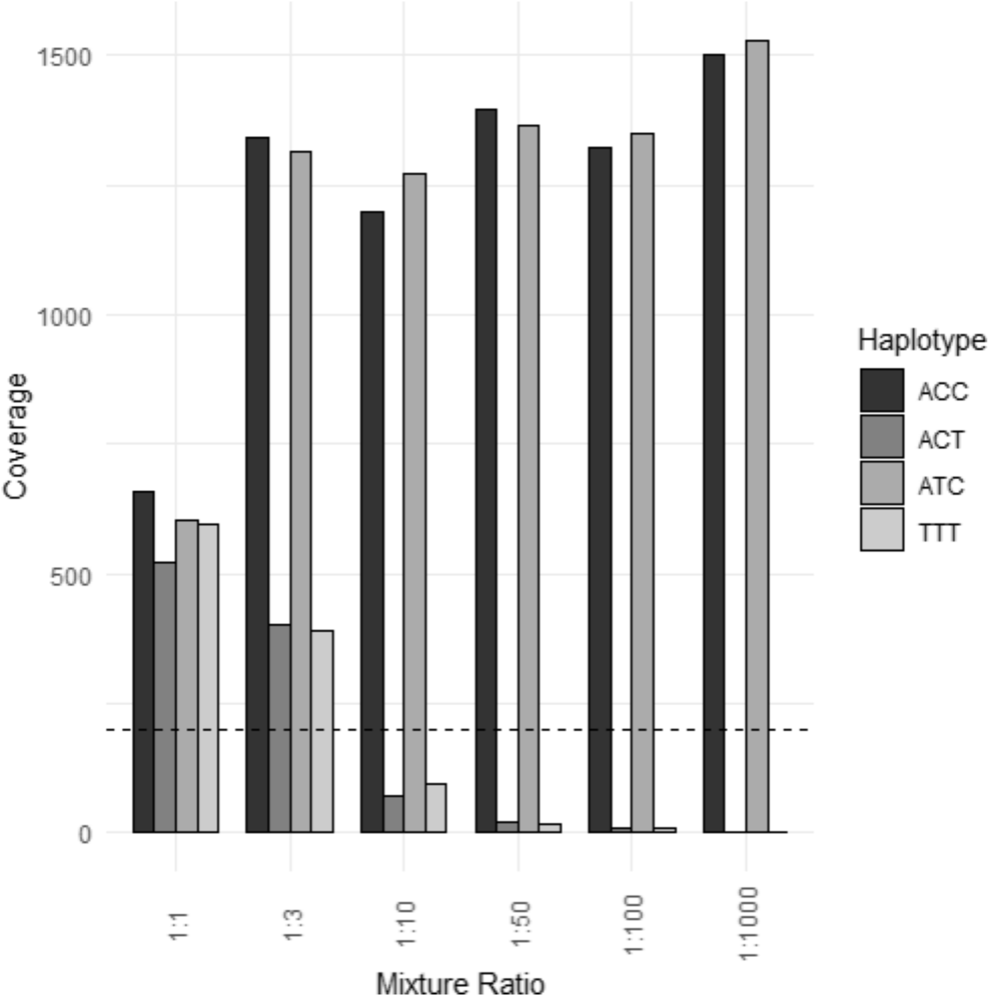
A boxplot of the read coverage for the different mixture ratios. The dashed line represents the user-defined threshold of 200 reads. The 1:3 ratio can easily be detected; however, we can distinguish the ratio pattern for the mixtures down to 1:100 although the read coverage is relatively low for those mixtures

The artificial DNA mixtures were analysed in duplicate for the six different mixture ratios (2800M:007). One marker, MH05, was chosen for evaluation since this was the only marker that displayed four different haplotypes for the two analysed samples. The haplotype combinations for the two samples were ACT, TTT for 2800M (Promega) and ACC, ATC for DNA control 007 (Thermo Fisher Scientific). For this marker, we could detect mixtures down to the 1:100 ratio with a median coverage of 9.5 reads for the minor contributor (median noise coverage was 1.5 reads). However, if we apply the previously defined coverage threshold of 200 reads, only the 1:3 mixture was detectable. The results are illustrated as a box plot in Fig. 4. One should take into consideration that these results only reflect the mixture of two specific samples at one specific locus. Further interpretations and extrapolations drawn from these results should be made with caution.

### Kinship analysis

Likelihood ratio calculations were performed in two different families with known biological relationships. We tested the hypothesis that each parent is the parent of the child versus the hypothesis that the parent is unrelated to the child, for both duo and trio paternity/maternity cases. The LR for the duo cases of the two different families ranged from 3*10^5^ to 6*10^8^ for one family and from 5*10^5^ to 7*10^8^ for the other family. For the trio cases, the LRs ranged from 2*10^10^ to 1*10^12^ and 2*10^12^ to 2*10^14^ for respectively family. See Supplementary file 5 for the LR per case.

**Fig. 5:**
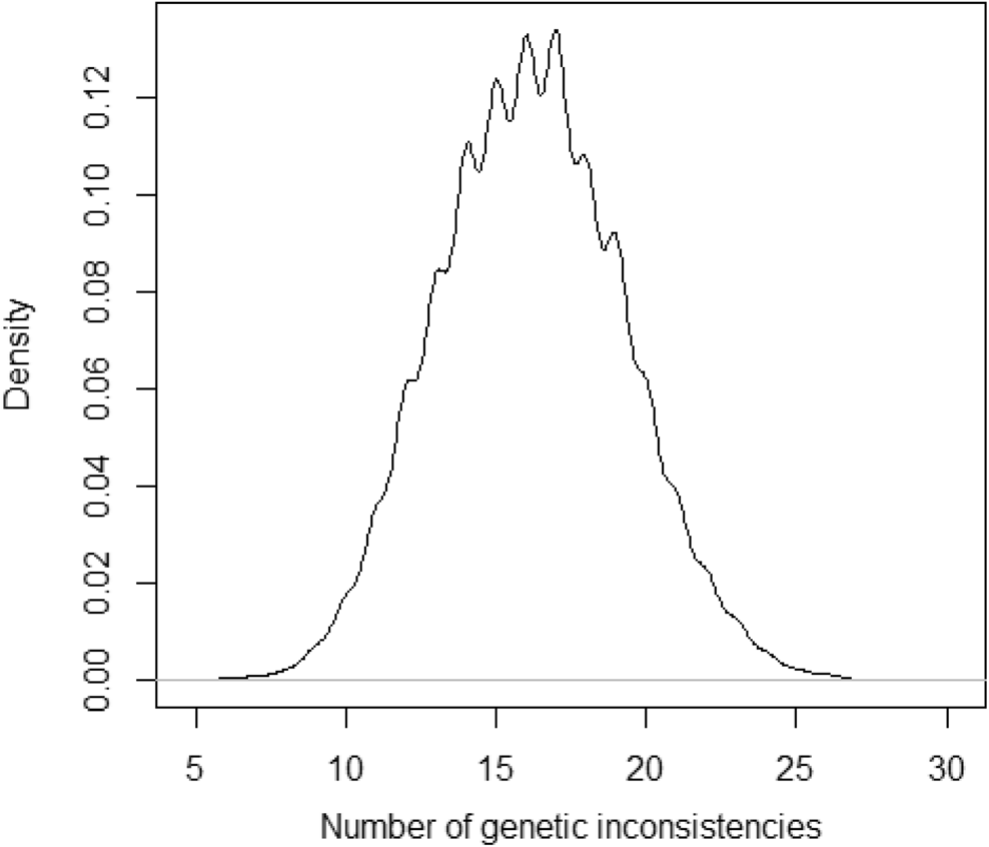
The number of genetic inconsistencies from 10,000 simulations of trio paternity tests is presented. The quantity ranges from 8 to 24 with an average of 16 markers

To further evaluate the discrimination power of the microhaplotype panel, we calculated the number of genetic inconsistencies when a man, unrelated to the child, was tested as the alleged father in trio paternity cases from 10,000 simulations based on the established microhaplotype frequencies. The simulation tested the hypothesis that an alleged father is the father of a child (hypothesis H1) versus that he is not the father of the child (hypothesis H2). Figure 5 displays a density plot with the number of genetic inconsistencies when hypothesis H2 is true, which shows that on average, around 16 genetic inconsistencies exist between the alleged father and the tested child. This could be considered as enough genetic inconsistencies to rely on the result as a true exclusion of the hypothesis that the tested man is the biological father of the child. Furthermore, the lowest number of genetic inconsistencies in a single case was eight which was observed in 11 of the simulated cases. These results strengthen the power of the panel and the risk of false inclusions can be considered as very low.

**Fig. 6:**
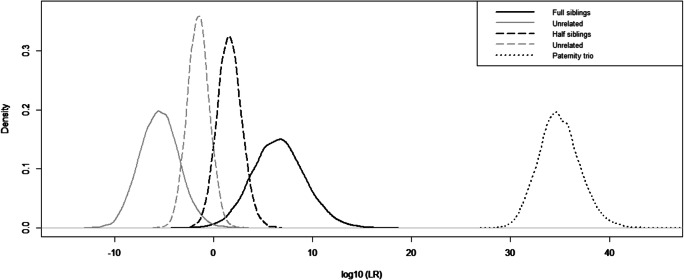
Distribution curves of LR for three tested relationships from 10,000 simulations are presented. The black lines show the LR for hypothesis H1 to be true and the grey lines represent the alternative hypothesis H2 to be true. The solid lines demonstrate the full sibling versus unrelated simulation, the dashed lines show the half sibling versus unrelated simulation and the dotted line represents the paternity trio versus maternity duo case. The full sibling simulation shows a clear separation of the hypothesis while there is a small overlap in the half sibling simulations

Furthermore, we performed 10,000 simulations in ILIR [27] and the result is illustrated in Fig. 6 as distribution curves for the LR for the three different case scenarios. For the full siblings versus unrelated scenario, the distribution curves are separated from each other and the risk of misinterpretations is therefore very low. In contrast, the half sibling versus unrelated case scenario has overlapping distribution curves. The area under this intersection point could cause false-positive and false-negative conclusions. Therefore, interpretation of these results should be made with caution and to avoid any incorrect conclusion, one could report these cases as inconclusive. Before implementing this method, it is vital to validate what range of LRs that should be considered as inconclusive. For the trio paternity case, the LR is in the order of 10^34^. The alternative hypothesis to the trio paternity case, a duo maternity, is not illustrated in Fig. 6, since no LR was generated for this hypothesis from the simulation. Instead, Fig. 5 represents this alternative hypothesis where the number of genetic inconsistencies is presented. However, one can conclude that the power of the panel for paternity tests is very strong based on the high LR for hypothesis H1 and the high number of genetic inconsistencies for hypothesis H2.

### Statistical parameters

One of the forty-four examined loci (MH23) showed a statistically significant departure from Hardy Weinberg equilibrium (HWE) expectations (*p* value <0.05). However, none of the loci significantly deviated from HWE after the Bonferroni correction. Linkage disequilibrium (LD) between the markers was examined and out of 946 pairwise comparisons, 53 displayed departure from equilibrium with *p* values <0.05. After Bonferroni correction, none of the pairwise comparisons displayed any deviation from linkage equilibrium.

The effective number of alleles was calculated for each of the analysed microhaplotype markers and is presented in Supplementary file 1. The *A*_e_ values ranged from 1.37 to 5.36, although a majority (27 markers) had an *A*_e_ less than 3. It has been shown that microhaplotype markers with an *A*_e_ larger than 3 are desirable for both lineage identification and mixture deconvolution [28]. This indicates that an increased *A*_e_ for some loci could result in additional lineage information. From this study, we have shown that paternity and full siblings can be properly assigned. For more distant relationships such as half siblings, a more powerful marker panel would however be desirable in order to obtain sufficient information. For this examined panel, one way to achieve a higher *A*_e_ at some markers would be to optimize the primer design to allow the inclusion of more SNPs, at already existing regions, which were not covered in this design.

The observed heterozygosity ranged from 0.269 to 0.813 and had an average value of 0.620. The value for each locus is presented in Supplementary file 1. In previous screening studies of microhaplotypes [28], the authors set a heterozygosity threshold of larger than 0.4 to include the microhaplotype in the panel. The result from this study showed that 41 of the 44 tested loci had a heterozygosity value >0.4. Supplementary file 6 illustrates the relation between heterozygosity and *A*_e_ as a scatterplot and we can observe an increase in the heterozygosity as the *A*_e_ increases.

## Conclusions

All except one of the tested microhaplotype loci in the assay showed coverage depth well above the user-defined threshold. The coverage was at the same time relatively balanced. We have shown that this panel is well functional for different input materials in both DNA amount and DNA quality. Furthermore, the kinship analyses showed that the use of the panel in paternity tests is very informative as well as to determine full sibling relations. The primer design was, however, not optimized and some regions were covered by more than two primer pairs resulting in inconclusive haplotype assignment. Due to this, a few markers had to be reduced in the number of individual SNPs which most probably also reduced the discrimination power of the panel. The aim of this study was broad and we have shown the potential of this panel in the different subprojects. However, the number of samples in some of the subprojects is quite small. Therefore, further analyses are required before implementing this panel in routine casework.

## Supplementary information

ESM 1 (XLSX 25.6 kb)

ESM 2 (XLSX 21.7 kb)

ESM 3 (PNG 165 kb)

High resolution image (TIFF 2257 kb)

ESM 4 (PNG 242 kb)

High resolution image (TIFF 1333 kb)

ESM 5 (XLSX 18.7 kb)

ESM 6 (PNG 31 kb)

## Data Availability

The datasets generated during and/or analysed during the current study are available from the corresponding author on reasonable request.
