# Supplementary figures and images for: Evaluation of microhaplotypes in forensic kinship analysis from a Swedish population perspective
Source: Int J Legal Med. 2021 Jan 28;135(4):1151–60. doi: 10.1007/s00414-021-02509-y (PMC8205927; doi:10.1007/s00414-021-02509-y)

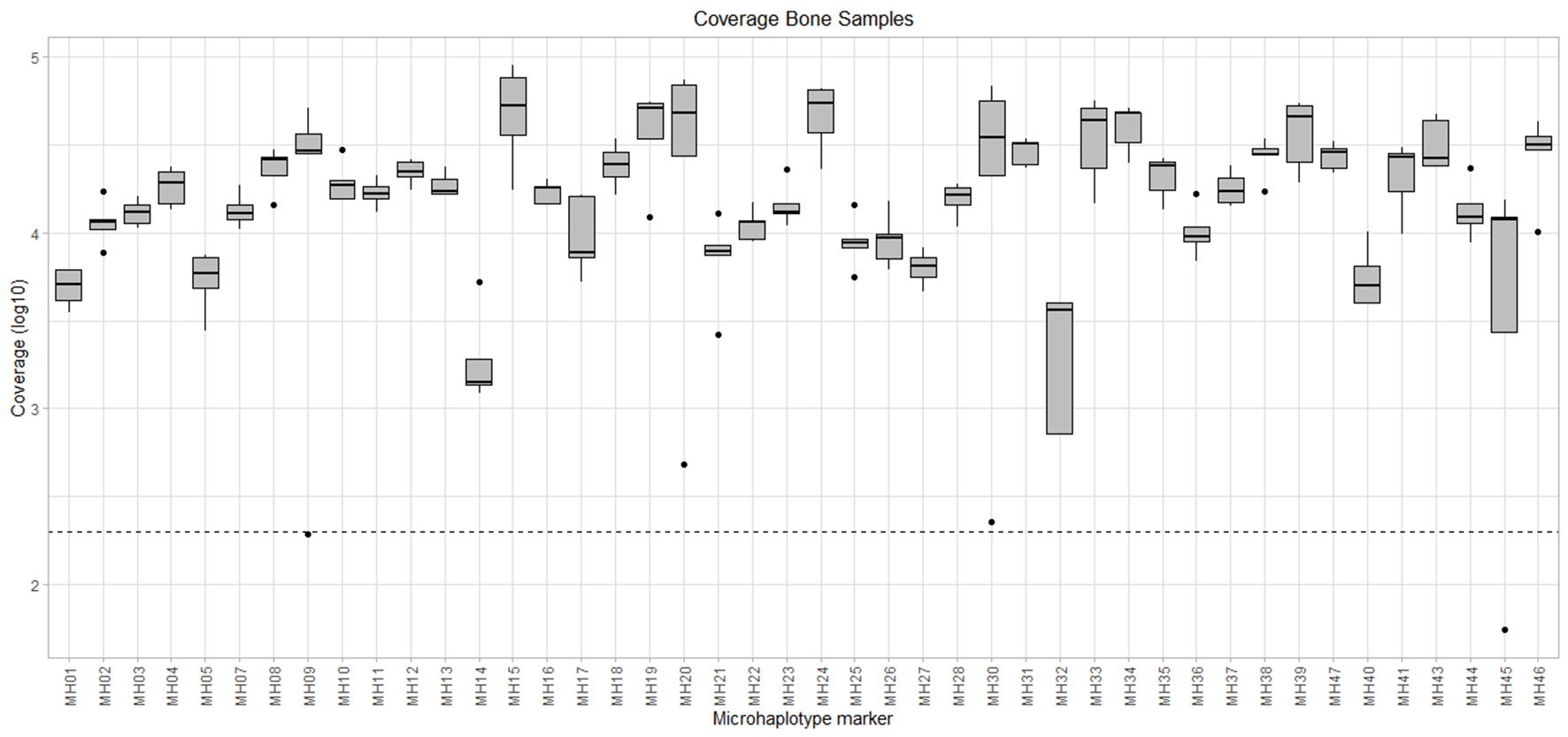

Supplement: Supplementary file 3 — (PNG 165 kb) [file 414_2021_2509_Fig7_ESM.png]

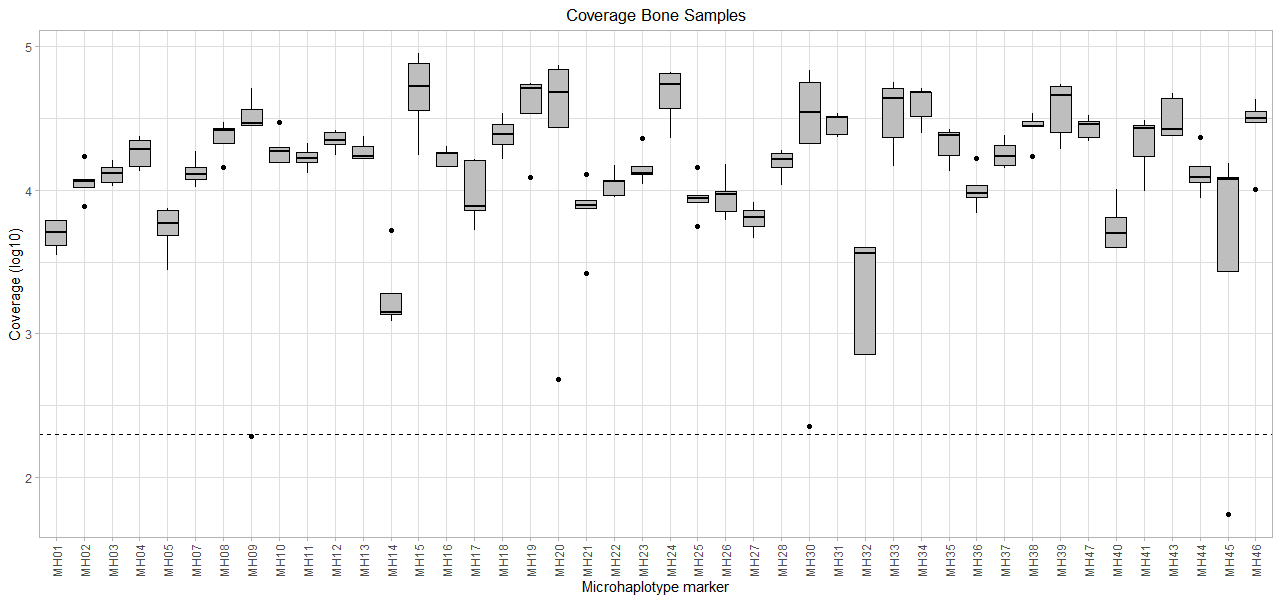

Supplement: Supplementary file 4 — High resolution image (TIFF 2257 kb) [file 414_2021_2509_MOESM3_ESM.tiff]

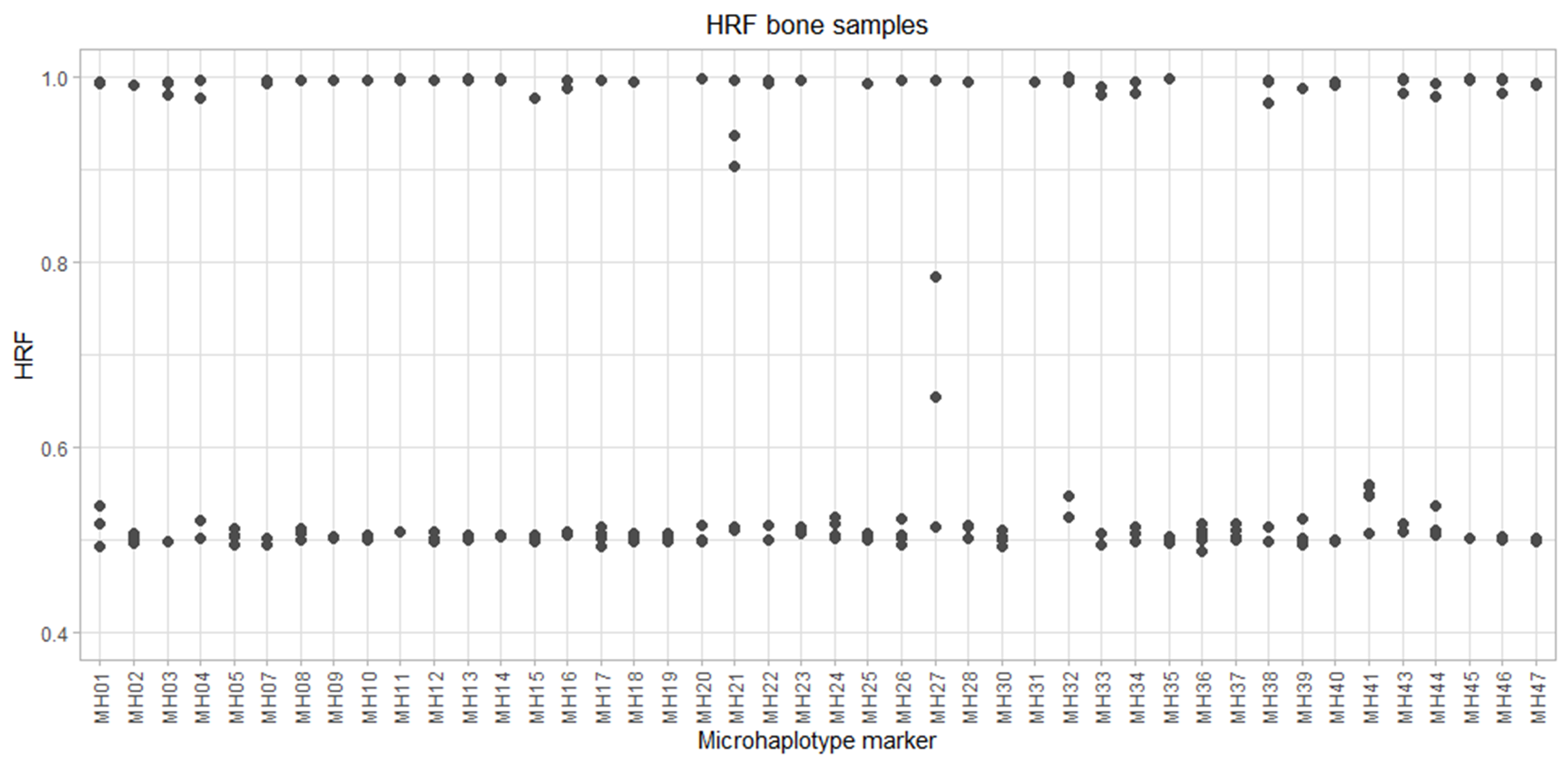

Supplement: Supplementary file 5 — (PNG 242 kb) [file 414_2021_2509_Fig8_ESM.png]

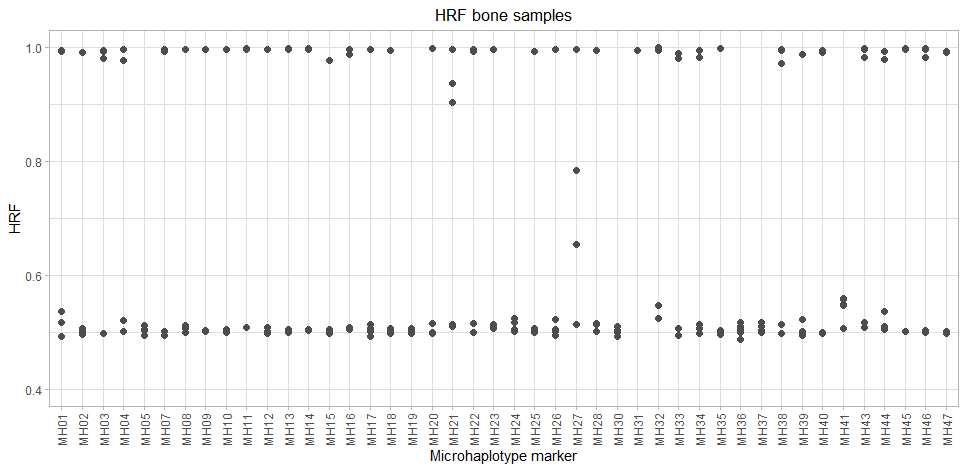

Supplement: Supplementary file 6 — High resolution image (TIFF 1333 kb) [file 414_2021_2509_MOESM4_ESM.tiff]

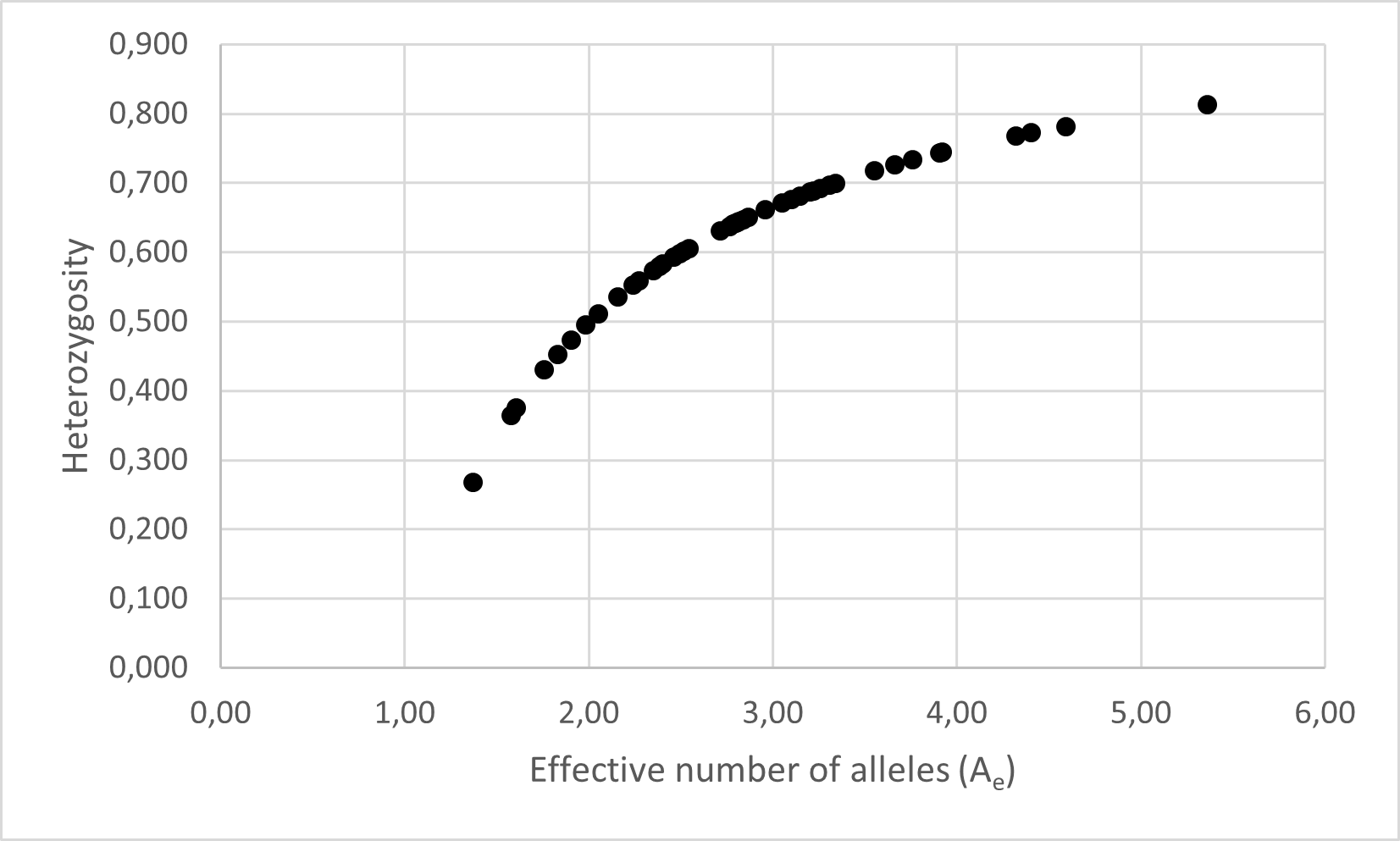

Supplement: Supplementary file 8 — (PNG 31 kb) [file 414_2021_2509_MOESM6_ESM.png]
